# Deconstructing the Conspiratorial Mind: the Computational Logic Behind Conspiracy Theories

**DOI:** 10.1007/s13164-022-00657-7

**Published:** 2022-08-27

**Authors:** Francesco Rigoli

**Affiliations:** Department of Psychology, City, University of London, Northampton Square, London, EC1V 0HB UK

## Abstract

In the social sciences, research on conspiracy theories is accumulating fast. To contribute to this research, here I introduce a computational model about the psychological processes underlying support for conspiracy theories. The proposal is that endorsement of these theories depends on three factors: prior beliefs, novel evidence, and expected consequences. Thanks to the latter, a conspiracy hypothesis might be selected because it is the costliest to reject even if it is not the best supported by evidence and by prior beliefs (i.e., even if it is not the most accurate). In this way, the model implies a key role for motivated reasoning. By examining the social conditions that favour the success of conspiracy theories, the paper embeds the model, whose focus is primarily psychological, within the broader social context, and applies this analysis to probe the role of conspiracy theories within contemporary Western societies. Altogether, the paper argues that a computational outlook can contribute to elucidate the socio-psychological dynamics underlying the attractiveness of conspiracy theories.

## Introduction

Conspiracy theories explain social and political events by claiming that a powerful group of people is damaging the own group or the whole community by means of secret plots.^1^ Research about the psychological processes underlying the appeal of conspiracy theories is accumulating fast (Butter and Knight 2020; Douglas et al. 2019; deHaven-Smith 2013; van Prooijen and Douglas 2018; van Prooijen and van Vugt 2018). Here I aim at contributing to this research by adopting computational modelling. The latter approach is relatively novel in social psychology, and yet, by relying on mathematical formalizations, it can offer unambiguous descriptions of the phenomena under scrutiny (Rigoli 2021a; Vallacher et al. 2017; Zmigrod and Tsakiris 2021). A computational outlook is especially well-suited to isolate the basic logic underlying a psychological process, thereby providing a platform to organize the theoretical and empirical knowledge available about that process.

The next section introduces the model, referred to as the Computational Model of Conspiracy Theories (CMCT). This is followed by a section embedding the model, whose focus is psychological, within the broader social context. Next, to provide an example of how a particular case can be analysed, the model is applied to interpret the role of conspiracy theories within contemporary Western societies.

## Psychological Processes

**Fig. 1:**
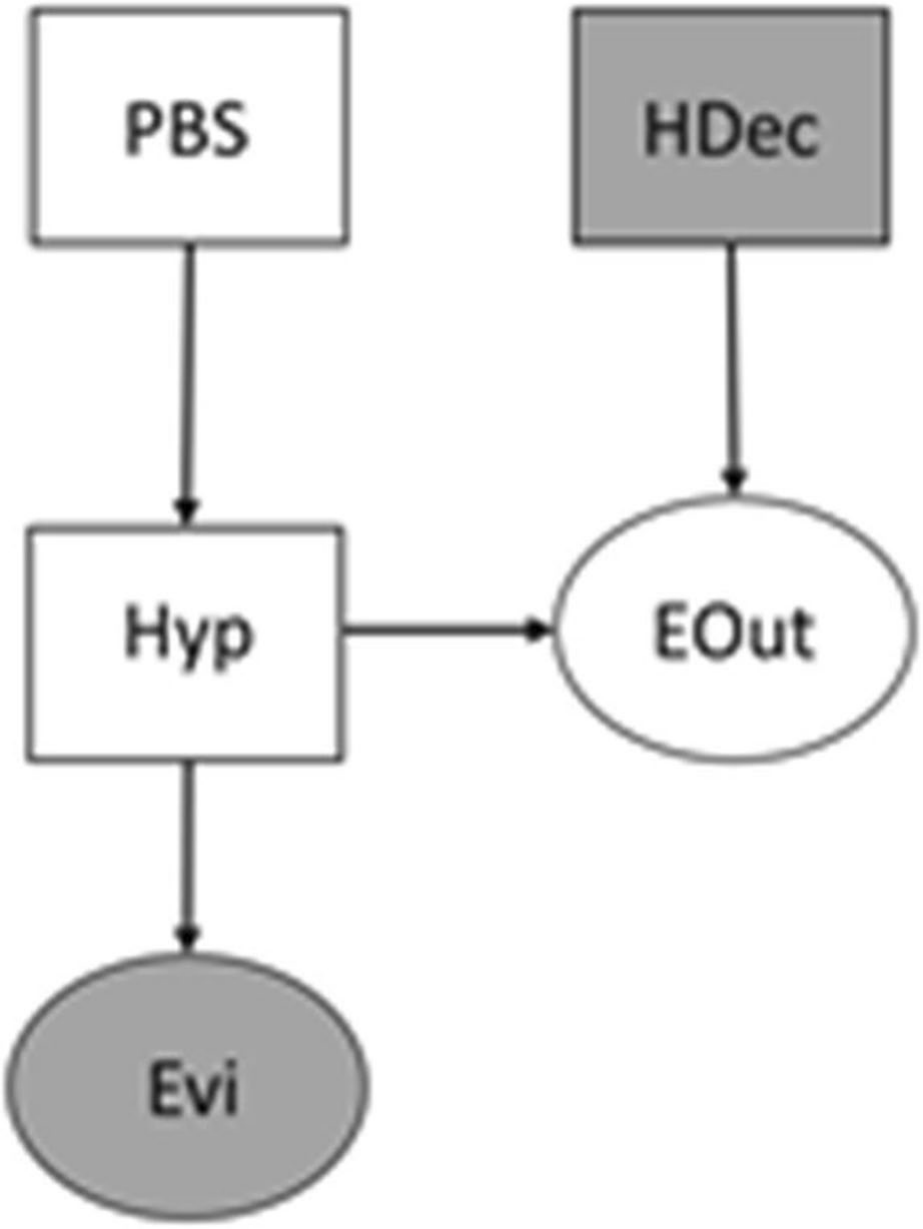
Bayesian network representing the CMCT. Its variables are: Prior Belief Systems (PBS), Hypothesis (Hyp), Evidence (Evi), Hypothesis Decision (HDec), and Expected Outcome (EOut). Categorical and continuous variables are represented by squares and circles, respectively. Arrows indicate probabilistic causal relations from one variable to another. Shaded variables are those considered to be observed at each step of the inference

**Fig. 2:**
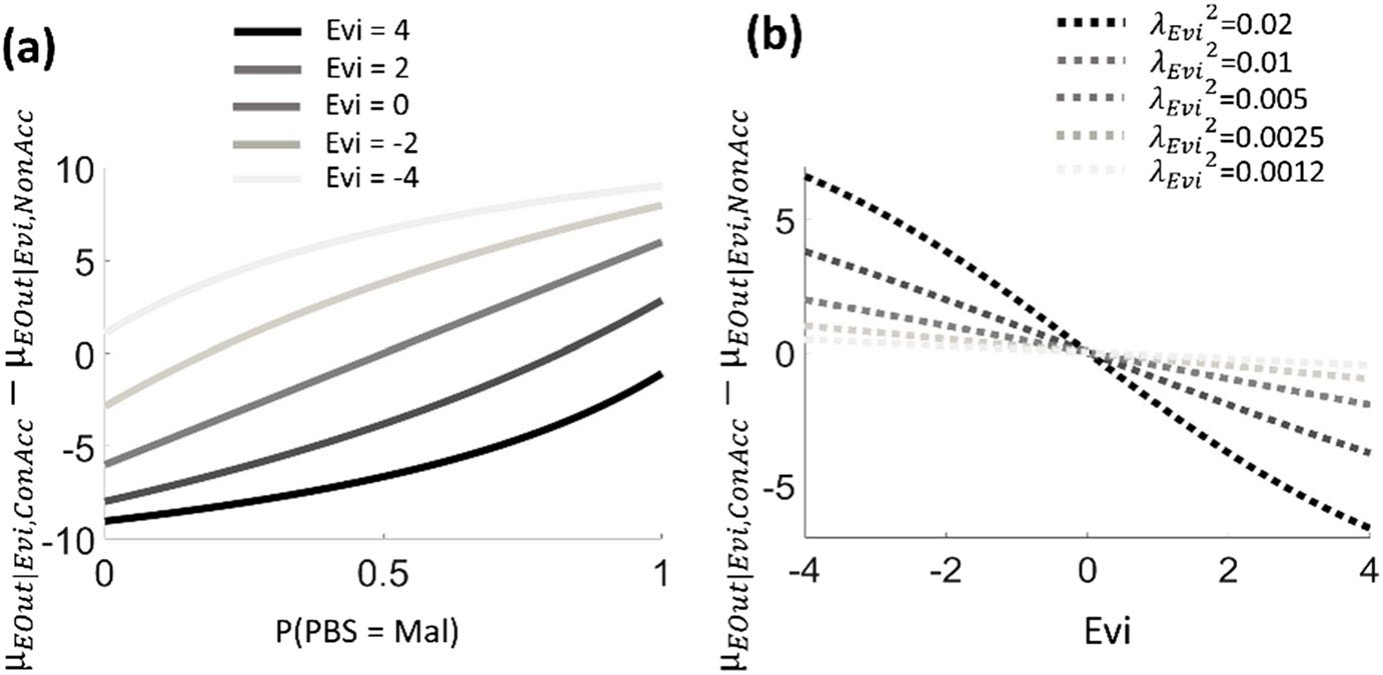
Simulation of the model. The simulated scenario is discussed also in the main text, where Hyp includes two categories (Conspiracy hypothesis vs Non-conspiracy hypothesis), PBS includes two categories (Malevolent (Mal) vs Honest (Hon)), and negative values of Evi support the Conspiracy hypothesis. The y axis reflects the expected consequences of accepting the Conspiracy hypothesis minus the expected consequences of accepting the Non-conspiracy hypothesis. **A**: The x axis reflects the prior probability for PBS = Mal. Different lines indicate different values for Evi (for all lines, the precision parameter for Evi is ${{\lambda }_{Evi}}^{2}=0.005$, the outcome of accepting the Conspiracy hypothesis when it is true (${\mu }_{EOut|Con,ConAcc}$) is equal to zero, the outcome of accepting the Non-conspiracy hypothesis when it is false (${\mu }_{EOut|Con,NonAcc}$) is equal to -10, the outcome of accepting the Non-conspiracy hypothesis when it is true (${\mu }_{EOut|Non,NonAcc}$) is equal to zero, the outcome of accepting the Conspiracy hypothesis when it is false (${\mu }_{EOut|Non,ConAcc}$) is equal to -10). **B**: The x axis reflects the value of Evi. Different lines indicate different values for the precision parameter for Evi ${{\lambda }_{Evi}}^{2}$ (for all lines, P(PBS = Mal) = 0.5 and other parameters are as above)

The CMCT builds upon a recent theory of motivated political reasoning (Rigoli 2021b, c; Rigoli et al. 2021) which examines how the brain arbitrates among competing hypotheses about social and political events. The theory can be implemented by a computational model based on a Bayesian decision framework (Bishop 2006) (see Appendix for details). Figure 1 describes the model adopting the formalism of Bayesian networks (Bishop 2006). The assumption is that the brain entertains beliefs about (i) salient variables (each associated with a probability distribution), represented by circles (for continuous variables) or squares (for categorical variables), and (ii) about their probabilistic relationships, represented by arrows. The first variable in the model is Hypothesis (Hyp), capturing competing interpretations of an important issue under consideration. In the CMCT, Hyp concerns cases where one conspiracy hypothesis is opposed to one (or more) non-conspiracy hypothesis. I will focus on an example where the conspiracy hypothesis claims that “a secret international clique of oligarchs has developed COVID-19 vaccines as a means to reduce people’s fertility and diminish the world population” whereas the non-conspiracy hypothesis argues that “COVID-19 vaccines are genuinely aimed at protecting people and do not impair fertility” (Stein et al. 2021). According to the CMCT, three factors are critical to establish which hypothesis will be endorsed by an individual. The first factor is represented by prior beliefs, indicating a set of assumptions already available before reasoning. In the CMCT, these are described by the variable Prior Belief System (PBS) (Fig. 2A); as the arrow projecting from PBS to Hyp indicates, PBS is assumed to influence Hyp. The impact of prior beliefs upon the attractiveness of conspiracy theories is supported by evidence showing that, while some people rarely rely upon conspiratorial interpretations, other people tend to express conspiratorial views over a variety of issues (Bruder et al. 2013; Dyrendal et al. 2021; Goertzel 1994). In the CMCT, this occurs because, for some people, prior beliefs embody a general mindset that favours interpretations of events as conspiratorial, independent of their content. Prior beliefs can capture assumptions about the personal sphere or about the general socio-political domain. Regarding the latter, PBS might include a view that political elites rarely plot against lay people versus the view that political elites constantly engage in conspiracies. The CMCT predicts that a conspiracy hypothesis will be more appealing for individuals assigning higher prior probability to the second view. As an example within the personal domain, PBS might oppose a view that people are generally honest versus the alternative view that people are malevolent. According to the CMCT, people assigning higher prior probability to the second view will be more likely to embrace a conspiracy hypothesis (Fig. 2A). This fits with evidence showing that conspiracy theories prosper among people with paranoid ideation (Darwin et al. 2011; Imhoff and Lamberty 2018) and with low levels of trust (Abalakina-Paap et al. 1999; Goertzel 1994). Another characteristic of prior beliefs is that, generally, they favour simpler hypotheses. This predicts that, other things being equal, conspiracy theories will be particularly attractive when they are simple (though sometimes conspiracy theories appear to be highly convoluted; according to the CMCT, factors other than prior beliefs are key in this latter case – see below).

The CMCT argues that the second critical factor affecting arbitration between hypotheses is represented by novel available evidence, which can be experienced in two ways: directly, when evidence is conveyed by the own perception, or socially, when social sources such as another person or the media provide indirect information. Within the CMCT, novel evidence is captured by the variable Evidence (Evi), represented by a continuous variable (with negative and positive values supporting the conspiracy and the non-conspiracy hypothesis, respectively). In our example, experiencing side effects following COVID-19 vaccination might be interpreted as direct evidence in favour of the conspiracy hypothesis (Fig. 2B). Although, for the sake of simplicity, one single variable Evi is included in Fig. 1, multiple Evi variables can be added to the model. The CMCT proposes that each Evi is linked with a specific level of reliability, formally captured by a precision parameter (see Appendix). This entails that information from unreliable sources (e.g., newspapers considered to be biased) will be dismissed, whereas information from reliable sources (e.g., newspapers appraised as unbiased) will be determinant for arbitrating between hypotheses. An implication of this is that different media sources are predicted to impact differently upon the success of conspiracy theories: media judged as reliable will be influential, while media appraised as untrustworthy will be ignored. This fits with the observation that people who are suspicious about institutional media are more likely to embrace “unofficial” conspiracy theories (Imhoff et al. 2018).

**Fig. 3:**
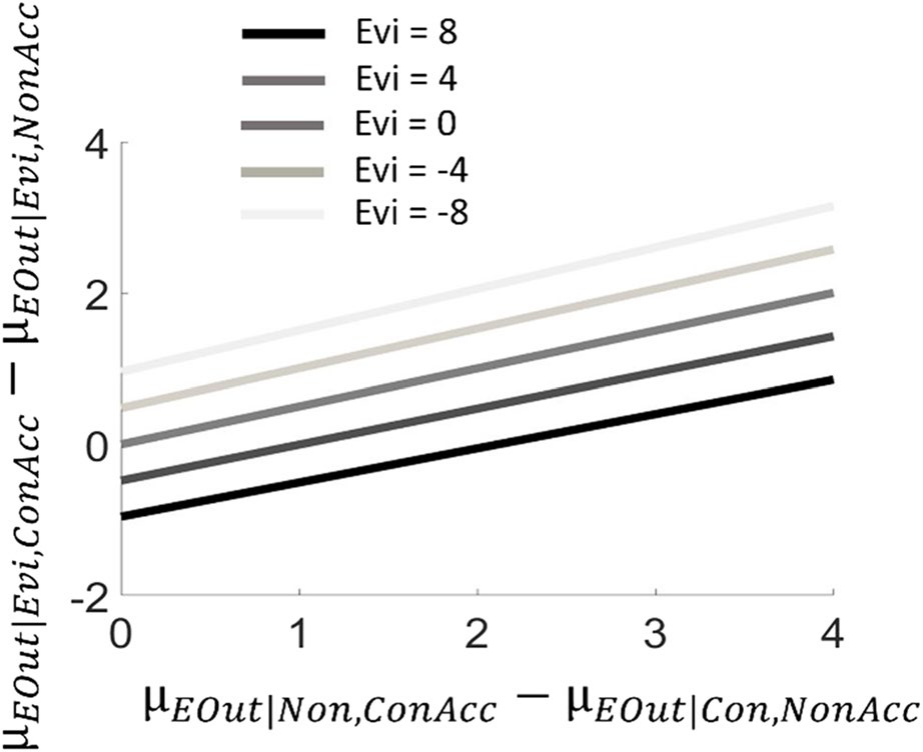
Simulation of the model about expected outcome (see main text and Fig. 2 for description of the scenario). The y axis reflects the expected consequences of accepting the Conspiracy hypothesis minus the expected consequences of accepting the Non-conspiracy hypothesis. The x axis reflects the difference between the expected outcome of accepting the Conspiracy hypothesis when it is false (${\mu }_{EOut|Non,ConAcc}$) and the expected outcome of accepting the Non-conspiracy hypothesis when it is false (${\mu }_{EOut|Con,NonAcc}$). Different lines indicate different values for Evi (for all lines, P(PBS = Mal) = 0.5, the precision parameter for Evi ${{\lambda }_{Evi}}^{2}=0.0012$, the expected outcome of accepting the Non-conspiracy hypothesis when it is true (${\mu }_{EOut|Non,NonAcc}$) is equal to zero, the expected outcome of accepting the Conspiracy hypothesis when it is false (${\mu }_{EOut|Non,ConAcc}$) is equal to -10, the expected outcome of accepting the Conspiracy hypothesis when it is true (${\mu }_{EOut|Con,ConAcc}$) is equal to zero)

The CMCT maintains that the third factor critical for arbitrating between hypotheses is represented by the consequences (in terms of reward or punishment) expected if any hypothesis is accepted or rejected (Rigoli 2021a, b; Rigoli et al. 2021). Formally, this is captured by the inclusion of the variables Hypothesis Decision (HDec) and Expected Outcome (EOut), the former being categorical (with categories being accepting the conspiracy hypothesis versus accepting the non-conspiracy hypothesis) and the latter being continuous (with positive numbers indicating reward and negative numbers indicating punishment). As Fig. 1 illustrates, EOut is assumed to depend jointly on Hyp and HDec. To examine how expected consequences exert their influence, let us look at how the CMCT arbitrates between hypotheses in some detail (see Appendix). The proposal is that, to adjudicate between the conspiracy and non-conspiracy hypothesis, the graphical model depicted in Fig. 1 is employed to predict the consequences of accepting each hypothesis (Fig. 3). Formally, this consists in estimating the posterior probability of EOut given Evi and HDec (P(EOut | Evi, HDec)). This inference is repeated for each hypothesis available (each time considering a different value of HDec as observed). Intuitively, this inference corresponds to assess the consequences expected to occur (i) if the conspiracy hypothesis is true and is accepted (and the COVID-19 vaccine is avoided), (ii) if the non-conspiracy hypothesis is true and is accepted (and the COVID-19 vaccine is taken), (iii) if the conspiracy hypothesis is false but is accepted (and the COVID-19 vaccine is avoided), (iv) if the non-conspiracy hypothesis is false but is accepted (and the COVID-19 vaccine is taken). Once the consequences of accepting each hypothesis are predicted, the CMCT proposes that the hypothesis associated with better expected consequences is the one which is accepted and believed to be true. Thus, in this process, expected consequences (captured by EOut) play a key role. To understand why, compare two people: a young woman desiring to have children soon, and an older woman with many grandchildren to care about. Compared to the older woman, for the younger woman accepting the conspiracy hypothesis (and avoiding vaccination) might appear as less risky than accepting the non-conspiracy hypothesis (and being vaccinated) because of her desire to have children and because of her young age (which diminishes potential risks associated with the illness; Zhai et al. 2020). Thus, other things being equal, the CMCT predicts that the young woman will be more likely to accept the conspiracy hypothesis. In this way, the CMCT implies a role for motivated reasoning (Douglas et al. 2017): it proposes that conspiracy theories are more likely to be accepted when the risk of rejecting them is perceived to be higher. Motivated reasoning is proposed to act subconsciously: people endorsing conspiracy theories would be convinced that their judgements are grounded solely on evidence, and not on considerations about expected consequences. In our example, the young woman might be staunchly convinced that the COVID-19 vaccine endangers fertility, without realising that this belief does not arise from a disinterested analysis of reality.

The role of expected consequences postulated by the CMCT offers an interpretation of empirical evidence showing that anxiety and lack of control encourage support of conspiracy theories (Bruder et al. 2013; Douglas et al. 2017; Grzesiak-Feldman 2013; Kofta et al. 2020). To understand why, consider again the example of the young woman greatly desiring to have kids. Imagine that, despite repeated attempts, the woman has so far failed to have kids, thus feeling anxious and powerless. According to the CMCT (and thanks to the role of expected consequences), when comparing the conspiracy against the non-conspiracy hypothesis, the former reduces the woman’s anxiety and re-establish a feeling of control. This is because accepting the conspiracy hypothesis (claiming that the COVID-19 vaccine reduces fertility) allows the woman to avoid any risk of harming fertility.

Moreover, following the CMCT, the role of expected consequences implicates novel empirical predictions. A first prediction is that the attractiveness of conspiracy theories increases not only when their acceptance helps preventing harm (as typically assumed by previous literature), but also when it helps achieving gains. Although this prediction remains to be tested empirically, it appears to describe well some historical episodes. Take the accusation made by the French king Philip the IV against the Templars for their alleged conspiracy against Christianity (Jones 2017), which resulted in the dissolution of the Templars and, to the advantage of Philip the IV, in the confiscation of the Templars’ rich properties. Arguably, the king’s conspiracy theory was not much motivated by perceiving danger, but rather by the opportunity of loot.

Considering the role of expected consequences within the CMCT, a second prediction is that a bias exists towards hypotheses which are costlier to reject, and not (as commonly assumed by previous literature) towards hypotheses which are wished to be true. To understand this subtle point, consider again the example above describing a woman who greatly desires to have kids. Obviously, from the woman’s perspective, the hypothesis that COVID-19 vaccines are safe (i.e., the non-conspiracy hypothesis) is more desirable. Yet, according to the CMCT, the woman will not be biased towards this hypothesis, but towards the alternative (i.e., the conspiracy hypothesis claiming that COVID-19 vaccines impair fertility), even if this is less desirable. This occurs because the conspiracy hypothesis is costlier to reject: if the hypothesis turned out to be true, its rejection (leading to vaccination and potentially to diminished fertility) would be catastrophic.

The notion of expected consequences proposed by the CMCT has similarities with error management theory (Haselton and Buss 2000) as applied to the study of conspiracy theories (van Prooijn and van Vugt 2018). This previous proposal argues that, when exposed to appropriate cues, evolution has predisposed humans to detect conspiracies (van Prooijn and van Vugt 2018). The reason is that, in evolutionary terms, the cost of disregarding actual conspiracies would be much greater than the cost of believing in untrue conspiracies: people who were not biased towards conspiracy detection, the argument goes, did not have sufficient evolutionary fitness and thus perished, favouring people endowed with such bias. The ensuing prediction is that humans have a general bias towards interpreting events as conspiracies. Although this argument presents similarities with the CMCT, the latter proposes something quite different: it proposes that, every time a conspiracy hypothesis is considered, the brain calculates the cost of rejecting it and thereby decides whether to accept the theory or not. Put another way, in the CMCT the costs of rejecting conspiracy theories are calculated each time by the brain, and are not an implicit calculation of evolution. As a consequence, the CMCT does not imply any human bias towards detecting conspiracies (note that any such bias remains to be documented empirically). In other words, within the CMCT expectations about consequences do not inherently favour conspiracy against non-conspiracy hypotheses: in our example, the older woman might be biased in favour of the non-conspiracy hypothesis (because, for her, vaccination is more convenient) as much as the younger woman is biased towards the conspiracy hypothesis (because, for her, vaccination is less convenient).

Altogether, the CMCT proposes that the attractiveness of conspiracy theories depends on three factors: prior beliefs, novel evidence, and expected consequences. Because of the influence of the latter, a conspiracy hypothesis might be selected because it is the costlier to reject even though it is not the best supported by evidence and by prior beliefs (i.e., even it is not the most accurate). In this way, the CMCT implies a key role for motivated reasoning (Douglas et al. 2017; Kunda 1990). The CMCT proposes that people are largely unaware of this, and simply perceive their beliefs as factually true (Trivers 2011). Note that, nonetheless, according to the CMCT motivated reasoning is not the whole story: prior beliefs and novel evidence remain fundamental, because a conspiracy hypothesis will be less likely to be accepted if it is poorly supported by them. Now that the psychological processes postulated by the CMCT have been examined, the next section analyses how these processes are shaped by the social context.

## The Social Context

By looking at historical sources, it appears that conspiracy theories (and, for that matter, conspiracies themselves) are as old as any other theory. This is evident when reading reports from ancient historians, who often proposed conspiratorial interpretations of events. A famous example is the account written by the Roman historian Tacitus about the plot orchestrated by Sejanus (the leader of the Imperial bodyguard) against the Roman emperor Tiberius (Tacitus 2004). More or less plausible conspiracy theories have emerged in virtually all times and places, advocated by believers and opposed by sceptics. Despite their ubiquity, conspiracy theories have arguably prospered more in specific times and places than in others. Is it possible to identify social conditions that facilitate the spread of conspiracy theories? The CMCT can offer some insight on this question, by highlighting three social factors that might be important.

First, not surprisingly, the model implies that conspiracy theories will flourish in societies where actual conspiracies are recurrent. This derives from two aspects: first, conspiracies will produce traces that, within the CMCT, can be detected as direct evidence; second, repeated experience of conspiracies will shape prior beliefs in such a way that, eventually, conspiracies will be expected a priori. Although a systematic analysis of which social environments foster actual conspiracies is beyond the scope of the manuscript, three likely candidates are worth mentioning: (i) monarchies with poorly established succession rules (as in the ancient Roman and Ottoman empire; Howard 2017; Veyne 1997), (ii) societies where the civil society is culturally and economically strong but is repressed politically (as during the Bourbon Restoration in France (Pilbeam, 182); here conspiracies are often enacted by the civil society), (iii) intense geopolitical and ideological conflicts (as during the Cold war when superpowers repeatedly conspired to overthrow regimes supporting the other geopolitical side (Leffler and Westad 2010)).

According the CMCT, conspiracy theories will flourish not only when actual conspiracies occur, but also when similar events do, although these events are not, strictly speaking, conspiracies. In other words, the idea is that occurrence of surprisingly negative social events, even when not caused by any conspiracy, is likely to fuel conspiracy theories. To understand this, imagine an individual who attempts to arbitrate between the following hypotheses: a conspiracy hypothesis claiming that a clique of financiers is controlling the economy at the expense of all other people, versus a non-conspiracy hypothesis claiming that globalization enriches everyone. Consider what happens when the individual experiences the hardship of an economic crisis (a negative unexpected event) akin to the one erupted in 2008: the event fits much better with the conspiracy, compared to the non-conspiracy, hypothesis, leading the individual to accept the former hypothesis (even if it is actually untrue). Note that, as this example shows, the proposal is that negative events support conspiracy theories only when alternative hypotheses are unrealistic (as the hypothesis that globalization enriches everyone appears to be; Milanovic 2016; Piketty 2018)); if realistic alternatives are available, these will be able to account for negative events, preventing groundless conspiracy theories to develop. Put it simply, the CMCT predicts that unrealistic conspiracy theories flourish in societies where available alternative views are unrealistic.

According to the CMCT, expected consequences are a second factor affecting the spreading of conspiracy theories within a society: in a society where many people expect to obtain advantages or to avoid risks by endorsing a conspiracy theory, this theory is predicted to flourish. As mentioned above, a remarkable historical example of this is the accusation made by the French king Philip the IV against the Templars. The role of expected consequences appears also crucial to interpret the emergence of conspiracy theories against Jews, which have punctuated European history since the Middle Ages (Schama 2014). For instance, the blood libel was a recurrent (groundless) medieval accusation claiming that Jews murdered Christian children and used their blood in religious rituals (Dundes 1991). When, on a recurrent basis, these accusations re-emerged, Jews were not only mistreated but often also expropriated to the advantage of non-Jews. This aspect is in line with the role of expected consequences as proposed by the CMCT.

The CMCT argues that the role of expected consequences becomes particularly important when the values at stake associated with accepting or rejecting hypotheses are dramatic. To understand this, consider the outbreak of the European Black death in 1348, arguably a situation where values at stake were dramatic indeed. At that time, many blamed the Jews for the pandemic and massacred them (Schama 2014). Adopting the CMCT, I speculate that these people were assessing two hypotheses: “the Black death is Jews’ fault; thus, perhaps this illness can be defeated by attacking Jews” versus “the Black death is totally uncontrollable”. Besides assessing evidence for or against the two hypotheses, these people might have asked: what are the consequences of accepting the anti-Jews hypothesis if this is true (and Jews are attacked)? And if it is false (and Jews are not attacked)? In this scenario, the anti-Jews hypothesis might have appeared attractive to many: although poorly supported by evidence, this hypothesis offered a chance, albeit remote, to cope with the pandemic.

Stress is a factor that might enhance the values at stake associated with accepting/rejecting hypotheses, thus rendering the role of expected consequences particularly important. At the social level, this implicates that, during times of social (e.g., economical, political, cultural, or military) crises, many people experience higher stress and hence are more influenced by expected consequences. It follows that groundless conspiracy theories motivated by expected consequences (e.g., the blood libel) become more appealing during social crises (see van Prooijen and Douglas (2017) for a similar argument). This fits with historical data on anti-Semitic conspiracy theories: reports from the Middle Ages describe resurgence of these theories typically during famines or natural calamities (Schama 2014). Likewise, the flourishing of Anti-Semitic conspiracy theories in Nazi Germany is hard to explain without linking it with the Great economic Depression (Kater 2011), and the anti-communist paranoia of McCarthyism (often expressed in groundless conspiracy theories) can be fully understood only in the context of the fear of a Soviet attack (Schrecker and Deery 1994).

When considering the role of expected consequences, it is important to emphasise that different groups have different interests and aspirations. Therefore, based on expected consequences, one conspiracy theory might appeal to one group but not to another. The French revolution in its late phase, when different factions accused the others of orchestrating plots, appears to be a historical example of different groups embracing competing conspiracy theories because of different interests at stake (Campbell et al. 2007).

According to the CMCT, the third and last social factor affecting the spread of conspiracy theories concerns social evidence, especially as conveyed by the media. Regarding the role of the media in a society, a broad classification can be proposed by distinguishing among (i) societies where the media are under the control of an authoritarian regime (e.g., like in Nazi Germany (O'Shaughnessy 2009)), (ii) societies where the media are independent and largely institutional, namely managed by professionals such as journalists, experts, and academics (e.g., in the West immediately before the internet revolution), and (iii) societies where the media are independent and include both institutional and non-institutional media (e.g., blogs, small newspapers, and single individuals or small groups writing in social media), the latter managed by non-professional lay people (e.g., in contemporary Western societies) (Schroeder 2018). Arguably, societies where the media are controlled by authoritarian powers are the most favourable for the spreading of groundless conspiracy theories: in these societies, especially when under threat, central powers can easily propagate groundless conspiracy theories against imagined external enemies (e.g., against Jews in Nazi Germany (Herf 2008)). It is debatable whether societies with institutional media only or societies with both institutional and non-institutional media offer a more fertile ground for groundless conspiracy theories (Uscinski et al. 2018; Wood 2013); we will consider this debate in the next section.

In summary, the CMCT identifies three factors that determine the spread of conspiracy theories within a society: the prevalence of actual conspiracies (or at least of unexpected negative events), the presence of advantages for any social group associated with embracing a conspiracy theory, and the influence of the media. Can this picture offer any insight on the role of conspiracy theories as expressed in specific societies? As a case study, the next section addresses this question by focusing on contemporary Western societies.

## Conspiracy Theories in the West Today

To understand present-day conspiracy theories in the West, it is paramount to embed them in the context of contemporary Western societies. Since the Seventies, economic inequalities have increased sharply in these societies (Milanovic 2016; Piketty 2018). The causes of such increase remain debated, with globalization, the proliferation of information technology, and financial deregulation being among the factors often proposed. An analysis of the phenomenon reveals that growing inequality is primarily driven by a shrinking of the middle-class combined with a remarkable enrichment of the very well-off (Milanovic 2016; Piketti 2018). For many of the baby boom generation, a middle-class life was a realistic prospect, coming with a secure and well-remunerated job combined with wide-ranging social welfare services. This prospect has become progressively less realistic: job security has dropped dramatically, wages have stagnated, and the welfare state has decisively downsized. Importantly, the prevailing public debate has initially failed to recognise the unfolding of these processes, often proposing interpretations moving in exactly the opposite direction. For example, right before the 2008 economic crisis, many economists still predicted unprecedent economic opportunities for most people (Colander et al. 2009).

Following such social and economic dislocation, many Western countries have witnessed a surge in political conflict, manifested in the rise of anti-establishment parties (e.g., Trump’s Conservative Party) and programs (e.g., Brexit) (Norris and Inglehart 2019). This conflict often opposes social strata who have benefitted from the contemporary economic environment (urban, young, skilled, and mobile workers) against strata who have lost ground in this environment (non-urban, older, poorly skilled, and less mobile workers), with the former and the latter supporting pro-establishment and anti-establishment political movements, respectively (Norris and Inglehart 2019).

Bearing this social context in mind, the CMCT can now be applied to interpret the nature of contemporary conspiracy theories in the West. Remember that, as examined above, the CMCT identifies three factors affecting the spreading of conspiracy theories within a society: direct evidence, expected consequences, and social evidence (especially the media).

Regarding direct evidence, remember that the CMCT proposes that conspiracy theories prosper in societies where actual conspiracies are rife. There is no proof that, in the West, conspiracies are overall more common today than in the recent past. However, as outlined above, there is a widespread deterioration in the economic condition of many; thus, negative social events are arguably common experience for a substantial number of people. As discussed above, the CMCT argues that negative social events fuel poorly grounded conspiracy theories when alternative explanations of these events are unconvincing. Does this apply to the West today? In some cases, it is possible that prevailing narratives appear to many as inadequate to explain recent social developments, with the 2008 economic crisis being a case in point (Colander et al. 2009). Compared to the prevailing narratives, conspiracy theories might hence appear to many as better explanations of negative social events, thus flourishing.

According to the CMCT, expected consequences are a second key factor: the attractiveness of a conspiracy theory is proposed to increase when acceptance of the theory appears to be advantageous. Is the role of expected consequences more important in the West today? The CMCT suggests that this might be the case, because it argues that stress enhances the values at stake associated with accepting/rejecting hypotheses, and because stress appears to be on the rise in contemporary Western societies (Almeida et al. 2020) (possibly as a consequence of the deteriorating economy (Mucci et al. 2016)). This argument entails that conspiracy theories will be more appealing for social strata experiencing more stress (i.e., those who are struggling more in the current socioeconomic environment). This fits with evidence indicating a high prevalence of conspiracy theories among lower classes and among groups who perceive themselves as less powerful (Crocker et al. 1999; Uscinski and Parent 2014).

More generally, the CMCT implies that different social strata will have different interests at stake, with repercussions upon the role of expected consequences. For instance, compare one hypothesis claiming that the economy is controlled by a small clique of financiers that can be identified and eliminated, against another hypothesis claiming that the economy is a complex system hard to modify. The first hypothesis will appeal more to someone who wants to change the economy (i.e., someone who is struggling in the current socioeconomic environment), while the second hypothesis will appeal more to someone who wants to preserve the current economic system (i.e., one who is prospering in the current socioeconomic environment). In line with this, evidence indicates that anti-establishment conspiracy theories are more likely to be advocated by people who oppose the establishment in the first place (Wood and Gray 2019).

Finally, the CMCT proposes social evidence, and especially the media, as the third key factor determining the success of conspiracy theories in a society. In the last twenty-five years, the internet revolution has allowed the advent of non-institutional media in the West, now living side by side with well-established institutional media (Schroeder 2018). Has the proliferation of non-institutional media favoured or disfavoured poorly grounded conspiracy theories? One aspect to consider is that institutional media are managed by professionals whose training prevents them to manipulate information in a blatant fashion; conversely, people with no formal training in dealing with information are the lifeblood of non-institutional media. This observation has led some scholars to the conclusion that the surge of non-institutional media has spread groundless conspiracy theories (Knight 2002; Marcus 1999; Wood 2013). On the other hand, although a plurality of interests and positions is surely expressed by institutional media, on a broader scale these still represent a restricted segment of society (the proliferation of non-institutional media might partially arise as a reaction of social segments who do not feel represented by institutional media). Therefore, institutional media are at risk of supporting poorly grounded conspiracy theories (or, for that matter, poorly grounded non-conspiracy theories alike) that favour this restricted social segment (within the CMCT, this is the product of expected consequences). Applying this reasoning to contemporary Western societies, remember that these are characterised by a growing conflict between people who are thriving in the contemporary socioeconomic environment (supporting pro-establishment positions) and people who are straggling in this environment (supporting anti-establishment positions) (Norris and Inglehart 2019). This conflict appears to be expressed also at a mediatic level, where most institutional media tend to support pro-establishment ideas, while several non-institutional media encourage anti-establishment arguments (Norris and Inglehart 2019). In this scenario, a dialectic between institutional and non-institutional media might ultimately disseminate more realistic social theories: non-institutional media can debunk biases proposed by institutional media while institutional media do the opposite.

In summary, the CMCT offers a framework for interpreting the role of conspiracy theories in contemporary Western societies. The CMCT argues that a failure of prevailing narratives to explain contemporary socioeconomic crises is a key reason for the success of conspiracy theories; the latter might appear to many as being better equipped for interpreting such crises. Moreover, expected consequences are proposed to play a key role, especially for highly stressed people (with the number of such people on the rise). Finally, the CMCT offers an interpretation of the role of the media, highlighting how expected consequences might be important in this context too. The argument is that a coexistence of institutional and non-institutional media might ultimately promote more realistic social theories.

## Discussion

The paper introduces the CMCT, a computational framework that analyses the psychological mechanisms underlying the success of conspiracy theories. This framework identifies prior beliefs, novel (social and direct) evidence, and expected consequences as key factors in the formation of conspiracy theories. Moreover, the paper embeds the CMCT within the social context and identifies social conditions which determine the success of conspiracy theories within a society, including the presence of conspiracies (and similar negative social events), the advantages associated with accepting conspiracy theories for different social groups, and the role of the media. This analysis is finally applied to explain the nature of conspiracy theories in the West today.

The CMCT can be extended further to explore aspects not considered in this paper. First, I have not examined how the generative model described in Fig. 1 is acquired in the first place. In other words, I have not addressed questions such as: why does one attribute higher prior probability to a conspiracy theory over another? Why are some sources considered more reliable than others? These are all crucial questions that remain to be explored within the CMCT. This exploration can benefit from considering previous literature adopting a Bayesian approach, such as literature examining the role of source reliability (Bovens and Hartmann 2003; Hahn et al. 2009; Harris et al. 2015; Madsen et al. 2020). Moreover, the CMCT might contribute to understand how to debunk false conspiracy theories and to prevent their success (Basol et al. 2021; Compton et al. 2021; Connor et al. 2020; Jolley and Douglas 2017). In this context, the CMCT stresses that it is crucial to consider the role of expected consequences, for example by identifying which social incentives boost the appeal of false conspiracy theories. Another extension of the CMCT consists in implementing a hierarchical generative model where prior beliefs over the priors themselves are included; in other words, a generative model characterised by a representation of confidence about priors (Behrens et al. 2007; Mathys et al. 2011). Considering such hierarchical architecture is useful to explore to what extent prior beliefs can be updated by novel experience. This aspect is interesting in the context of conspiracy theories: potentially, it can capture people’s tendency to fixate upon a false conspiracy theory even when this is disconfirmed by experience (Gershman 2019; Madsen et al. 2020).

Above, I have described hypothesis selection as an all-or-nothing process where one hypothesis is embraced while alternative hypotheses are discarded. However, to be precise, the CMCT offers a more nuanced picture: it posits that reasoning results in attributing a weight (formally, corresponding to the posterior expected consequences) to each hypothesis. In other words, despite one hypothesis being favoured over the others, different hypotheses are treated as being to some extent possible. This view applies also to conspiracy theories, in line with empirical evidence indicating that supporters of a conspiracy theory often remain open to alternative possibilities (even to alternative conspiracy theories; Wood et al. 2012).

In summary, the CMCT offers an intriguing perspective to interpret the basic psychological logic underlying the attractiveness of conspiracy theories. Besides being broadly consistent with available evidence, the model makes novel predictions that can inspire empirical research. Moreover, the CMCT offers a platform to integrate the psychological level of analysis (which is the main focus of the theory) with the social level. On this basis, an intriguing research avenue is to apply this framework to appraise the role of conspiracy theories in different times and places, as examined here in the context of contemporary Western society.

## Appendix

Let us examine formally the CMCT focusing on the example above where a conspiracy hypothesis such as “A secret international clique of oligarchs has developed COVID-19 vaccines as a means to reduce people’s fertility and, thus, to diminish the world population” is opposed to a non-conspiracy hypothesis such as “COVID-19 vaccines are genuinely aimed at protecting people and do not impair fertility”. Formally, the CMCT is a mixture of Gaussians. The joint probability can be written as:

$$P(PBS, Hyp, HDec, EOut, Evi)=P(PBS) P(HDec) P(Hyp|PBS) P(Evi|Hyp) P (EOut|Hyp, HDec)$$

PBS is a categorical variable with number of categories equal to ${n}_{PBS}$ and where each category is associated with a probability. In the example, we can set ${n}_{PBS}=2$, *PBS* = *Mal* if people are generally viewed as malevolent, and *PBS* = *Hon* if people are generally viewed as honest. The probability of people being malevolent is *P(PBS* = *Mal)* = *x* and the probability of people being honest is *P(PBS* = *Hon)* = *1—x* (where 0 ≤ *x* ≤ 1). Hyp is also categorical, with number of categories equal to ${n}_{Hyp}$. In our example, we can set ${n}_{Hyp}=2$, *Hyp* = *Con* for the conspiracy hypothesis, and *Hyp* = *Non* for the non-conspiracy hypothesis. The conditional probabilities for Hyp are *P(Hyp* = *Con | PBS* = *Mal)* = *y*, *P(Hyp* = *Non | PBS* = *Mal)* = *1 – y*, *P(Hyp* = *Con | PBS* = *Hon)* = *z*, *P(Hyp* = *Non | PBS* = *Hon)* = *1 – z* (where 0 ≤ *y* ≤ 1 and 0 ≤ *z* ≤ 1). HDec is also categorical, with the number of categories ${n}_{HDec}={n}_{Hyp}$. In our example, *HDec* = *ConAcc* when the conspiracy hypothesis is accepted (or, equivalently, when the non-conspiracy hypothesis is rejected) and *HDec* = *NonAcc* when the conspiracy hypothesis is rejected (or, equivalently, when the non-conspiracy hypothesis is accepted). Probabilities for HDec are *P(HDec* = *ConAcc)* = *u* and *P(HDec* = *NonAcc)* = *1—u* (where 0 ≤ *u* ≤ 1).

Evi is represented by a real number and follows a Gaussian distribution, with negative numbers supporting the conspiracy hypothesis and positive numbers supporting the non-conspiracy hypothesis. Evi is conditioned on Hyp, with conditional probability defined as:

$$\mathrm{P}(\mathrm{Evi}|\mathrm{Hyp}=\mathrm{k})=\mathcal{N}{(\mu }_{Evi|k}, 1/{{\lambda }_{Evi}}^{2})$$

Here, every category of Hyp *k* has its own associated average ${\mu }_{Evi|k}$; for instance the model includes ${\mu }_{Evi|Con}$ (conditional on the conspiracy hypothesis being true) which is different from ${\mu }_{Evi|Non}$ (conditional on the non-conspiracy hypothesis being true). The parameter ${{\lambda }_{Evi}}^{2}$ reflects the reliability or precision of Evi and in our model is equal for all levels of Hyp (in principle, a specific reliability for each level of Hyp can be implemented).

Finally, EOut is a Gaussian variable conditioned on both Hyp and HDec. Its conditional probability is:

$$\mathrm{P}(\mathrm{EOut}|\mathrm{Hyp}=\mathrm{k},\mathrm{ HDec}=\mathrm{j})=\mathcal{N}{(\mu }_{EOut|k,j}, {\sigma }_{Eout}^{2})$$

This indicates a specific average for each combination of Hyp and HDec. In our example, the model comprises ${\mu }_{EOut|Con,ConAcc}$ (the expected outcome if the conspiracy hypothesis is true and it is correctly accepted), ${\mu }_{EOut|Con,NonAcc}$ (the expected outcome if the conspiracy hypothesis is true but it is wrongly rejected), ${\mu }_{EOut|Non,NonAcc}$ (the expected outcome if the non-conspiracy hypothesis is true and it is correctly accepted), ${\mu }_{EOut|Non,ConAcc}$ (the expected outcome if the non-conspiracy hypothesis is true but it is wrongly rejected). The parameter ${\sigma }_{Eout}^{2}$ reflects the uncertainty about the outcome and in our model it is equal for all combinations of Hyp and HDec (although in principle one can also implement a specific uncertainty for each combination).

The model is used to make inference. For inference, the variable Evi is observed, while the other variables are not. The inference process includes multiple inference steps. At each step, for each level of HDec j, the model infers the conditional probability of EOut given the observed values for Evi and given HDec = j. This corresponds to a posterior Gaussian distribution:

$$\mathrm{P}(\mathrm{EOut}|\mathrm{Evi},\mathrm{ HDec}=\mathrm{j})=\mathcal{N}({\mu }_{EOut|Evi,j}, {\sigma }_{POST}^{2})$$

where ${\mu }_{EOut|Evi,j}$ is the posterior average for the expected outcome. In our example, ${\mu }_{EOut|Evi,ConAcc}$ will be the posterior average if the conspiracy hypothesis is accepted and ${\mu }_{EOut|Evi,NonAcc}$ is the posterior average if the non-conspiracy hypothesis is accepted.

After these inferences are made, the model makes a decision by choosing the hypothesis associated with the highest posterior ${\mu }_{EOut|Evi,j}$. In our example, it will either choose to accept or reject the conspiracy hypothesis (or, equivalently, to reject or accept the non-conspiracy hypothesis, respectively).

## Footnotes

1 while some scholars limit the term conspiracy theories to false theories only, in the paper I employ this term to describe theories that are false as well as theories that are true.
